# Iatrogenic aortic dissection in patients undergoing coronary artery bypass grafting surgery: A systemic review of published literatures

**DOI:** 10.1097/MD.0000000000037472

**Published:** 2024-03-22

**Authors:** Xi Yuan, Yanting Sun, Huaqiu Chen, Qiqing Lan, Wen Wu, Yuntai Yao

**Affiliations:** a Department of Anesthesiology, Xichang People’s Hospital, Xichang, China; b Department of Anesthesiology, National Center for Cardiovascular Diseases, Chinese Academy of Medical Sciences, Fuwai Hospital, Peking Union Medical College, Beijing, China; c Department of Anesthesiology, Baoji High-Tech Hospital, Shaanxi, China; d Department of Laboratory, Xichang People’s Hospital, Xichang, China.

**Keywords:** aortic dissection, case reports, coronary artery bypass grafting surgery, iatrogenic aortic dissection

## Abstract

**Background:**

Iatrogenic aortic dissection (IAD) is a rare but highly lethal complication that may occur following coronary artery bypass grafting (CABG) surgery. Aortic dissection (AD) is often asymptomatic, making early detection difficult. We aimed to optimize preoperative evaluation strategies of CABG surgery for minimizing the incidence of IAD and assess early recognition and management of IAD for improving outcomes.

**Methods:**

Electronic databases were searched to identify all case reports of patients undergoing CABG surgery who developed IAD. Clinical characteristics, operative information, perioperative management, and patient outcomes were compiled and analyzed.

**Results:**

Nineteen case reports involving 27 patients aged 50 to 81 were included. Patients were from Europe (n = 23) and Asia (n = 4), mostly men (n = 25). The aorta was described as normal, abnormal, and unmentioned (n = 8, 5, and 14, respectively). Sixteen patients had a bypass with more than 3 grafts. Most patients (n = 25) experienced type A dissection. There were intraoperative (n = 12) and postoperative (n = 15) cases. Surgery (n = 19) was the most common treatment, with 9 patients selecting deep hypothermic circulatory arrest. Eighteen patients were restored to health, while 9 patients died (3 died before treatment).

**Conclusions:**

Our study focused on patients with IAD and developed a recommended management protocol for patients undergoing CABG surgery.

## 1. Introduction

Aortic dissection (AD) is the formation of a layer within the wall of the aorta, which hinders blood flow and can potentially lead to aortic rupture and internal bleeding. Although iatrogenic aortic dissection (IAD) is highly uncommon, its potential fatality puts it at a severe risk.^[1]^ It should be noted that the incidence of IAD is higher compared to spontaneous AD. The surgical procedure itself carries a particular risk of arterial damage, and factors such as prolonged operation time, intraoperative arterial perforation, inadequate blood pressure control, and lack of proficiency in surgical techniques can further increase the risk of IAD. Once IAD occurs, it significantly increases the costs of hospitalization and length of hospital stay. Moreover, IAD may even lead to catastrophic outcomes. In coronary artery bypass surgery, surgeons implant a graft to restore blood flow in narrowed or blocked coronary arteries. However, this procedure itself can cause damage to the aortic wall, leading to the formation of a dissection. All at once, the proportion of coronary artery bypass grafting (CABG) in cardiac surgery is increasing annually because of the aging population and improvements in living standards.

A review of 27 case series involving 311 patients with IAD highlighted a concerning trend: 61% of these cases occurred during CABG surgery, while 12% occurred during aortic valve replacement surgery.^[2]^ This review analyzed and compared information on patients with IAD from multiple countries. With a large sample size, the research findings have a high reference value. Therefore, it provides a basis and inspiration for studying IAD induced by CABG. Remarkably, these percentages closely mirror those reported in surgeries performed in the United States, as indicated in the Society of Thoracic Surgeons database. Several factors during the surgical procedure may increase the risk of AD, particularly in patients with preexisting aortic pathology. The pathophysiology of AD following CABG surgery involves multiple factors such as atherosclerosis, surgical manipulation of the aorta, and hemodynamic stress. Compared to other cardiovascular surgeries, CABG carries a higher risk of inducing IAD. It is crucial to implement urgent strategies aiming at preventing the occurrence of IAD. However, it is worth noting that there are currently no specific guidelines for preventing IAD in the context of CABG procedures. We conducted a comprehensive literature review of published case reports to bridge this knowledge gap. Through this review, we aimed to summarize and analyze the clinical characteristics, operative details, perioperative management, and outcomes of patients undergoing CABG surgery.

By gathering and synthesizing information from these case reports, we hope to contribute to a better understanding of IAD in the context of CABG surgery. This knowledge can potentially direct the development of evidence-based guidelines and strategies to minimize IAD’s incidence and associated risks. Ultimately, our goal is to improve patient outcomes and promote safer and more effective CABG procedures.

## 2. Materials and methods

### 2.1. Search strategy

Relevant case reports were identified through computerized searches on PubMed and Web of Science databases until September 10, 2023, using different combinations of search terms, such as “aortic dissection,” “coronary artery bypass grafting,” “iatrogenic,” “cardiovascular surgery,” and “case reports.” The databases CNKI, Wanfang, and VIP were searched (from inception to September 10, 2023). Two authors (X.Y. and Y.T.Y.) independently reviewed the titles and abstracts of all identified reports to determine their eligibility and exclude those that did not meet the criteria. The final inclusion of the remaining studies was determined by examining their full texts. The exclusion criteria consisted of review articles, animal studies, duplicate publications, and studies lacking relevant outcomes.

### 2.2. Data abstraction

The following data from the included case reports were abstracted and tabulated by each author independently: literature information (author and year of publication); patient characteristics (age, sex, and medical history); information on CABG surgery (on-pump or off-pump and graft number); IAD features (occurrence time, type, diagnostic means, and treatment methods); and patient outcomes. Disagreements were resolved by discussion among all authors during data abstraction.

## 3. Results

**Figure 1.**
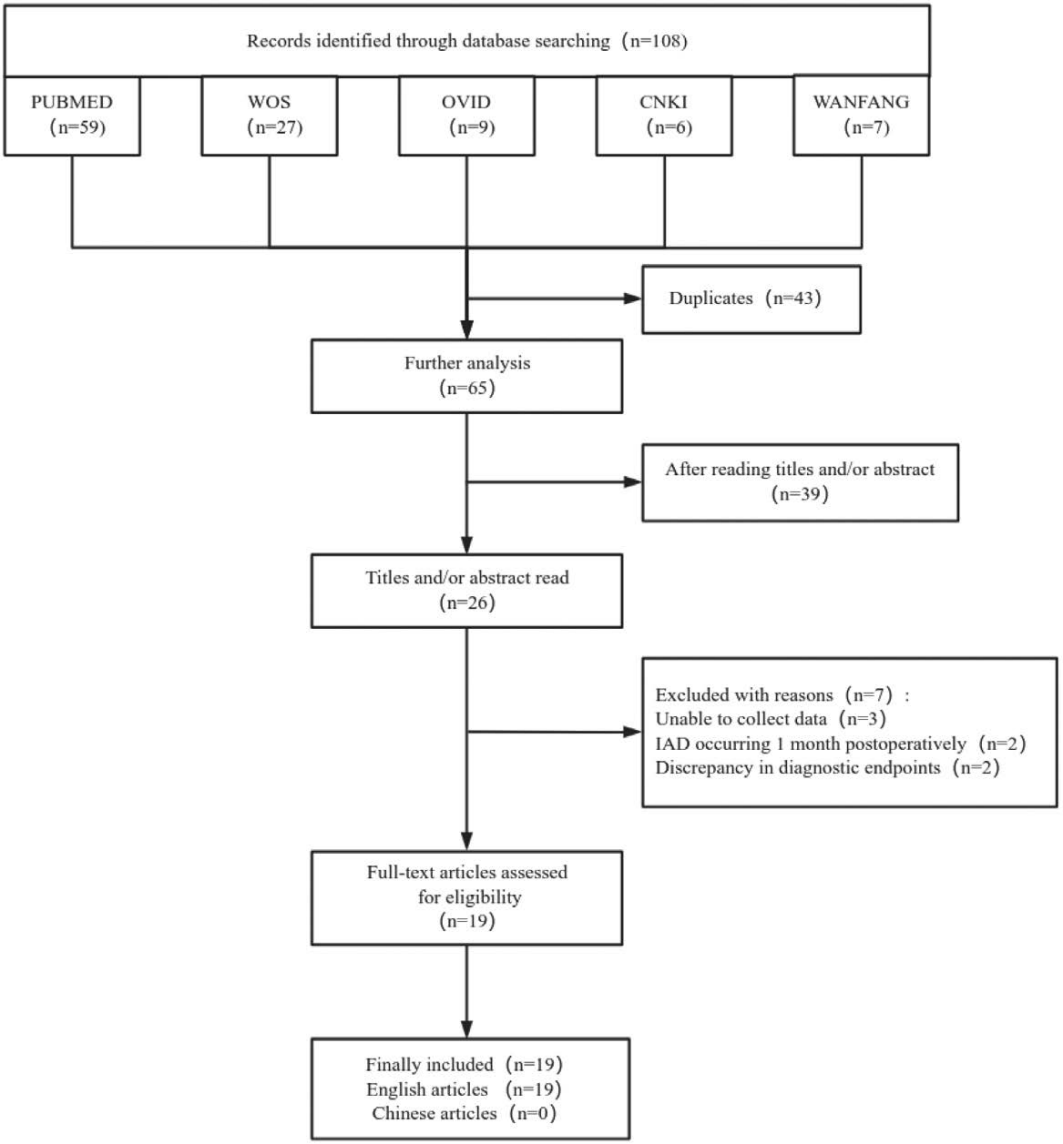
Flow chart.

According to the flowchart (Fig. 1), the database search identified 105 potentially eligible articles. After screening, 19 case reports involving a total of 27 patients were deemed suitable and included in the study, of which 17 were written in English. Table 1 presents the descriptive analysis of these cases.^[3–21]^

**Table 1:** General condition and characteristics of patients experienced IAD undergoing CABG surgery.

Author, year	Country	Age/sex	SAP?	Aortic	Medical history	On-pump?	Graft number	IAD				Treatment			Outcome
								Type	Diagnosis	Timing	Originates	Surgery?	Cannulation	CA?	
Chavano 2001^[3]^	Canada	70/M	Yes	/	HTN	No	3	A	CT-scan, TEE	POD13	Aortic side-clamping site	Yes	/	Yes	Death
	Canada	56/M	No	/	HTN	No	3	A	Macroscopic	IO	Aortic side-clamping site	Yes	Femoral-femoral	Yes	Discharge
	Canada	76/M	Yes	/	HTN	No	3	/	Autopsy	POD 5	Aortic side-clamping site	No	/	/	Death
	Canada	70/M	No	/	Obesity	Yes	3	A	Macroscopic	IO	Aortic side-clamping site	Yes	Femoral-aorta	/	Discharge
Assaad 2013^[4]^	America	63/M	No	Abnormal	HTN	Yes	1	A	Ultrasound	IO	Aortic cannulation site	Yes	Femoral-aorta	Yes	Discharge
Li 2017^[5]^	China	64/M	No	/	HTN, Gout	No	4	A	Macroscopic	IO	/	Yes	Right atrium – femoral	Yes	Death
Tabry 2009^[6]^	England	74/M	No	Normal	HTN	No	4	A	CT-scan	POD 7	Proximal anastomosis	Yes	/	/	Discharge
	England	61/M	No	Normal	HTN	No	3	A	CT-scan	POD 3	Proximal anastomosis	No	/	/	Death
	England	81/M	Yes	Normal	HTN	No	2	A	CT-scan	POD 20	Proximal anastomosis	Yes	/	/	Death
De 2003^[7]^	Belgium	70/M	No	/	HTN	No	2	A	CT-scan	POD 7	Proximal anastomosis	Yes	Femoral-femoral	Yes	Discharge
Takahashi 2021^[8]^	Japan	68/M	No	Normal	HTN, Dyslipidemia	No	3	A	CT-scan	POD 7	Posterior wall of aortic	Yes	/	/	Discharge
Ram 2020^[9]^	America	55/F	No	/	Dyslipidemia, Depression	Yes	4	A	TEE	IO	Aortic root	No	/	/	Discharge (ARF )
Pappas 1998^[10]^	America	57/M	No	Normal	/	Yes	/	A	TEE	POD 1	Aortic cannulation site	Yes	Bifemoral-axillary artery	/	Discharge
	America	68/M	No	/	/	Yes	/	A	TEE	POD 23	Aortic root	Yes	Femoral-femoral	/	Discharge
Archer 1986^[11]^	America	62/M	No	Abnormal	/	Yes	5	A	CT-scan	POD 0	Proximal anastomosis	No	/	/	Death
Cottrell 2003^[12]^	America	73/M	/	Normal	/	Yes	4	A	TEE	IO	Aortic cannulation site	Yes	Right atrium – aortic	Yes	Discharge
Bilgutay 1976^[13]^	America	57/M	No	Abnormal	/	Yes	1	A	Macroscopic	IO	/	Yes	Right atrium – femoral	/	Discharge
Magishi 2010^[14]^	Japan	77/M	/	/	/	No	3	A	TEE	IO	/	No	Right atrium – axillary artery	/	Discharge (BLP)
Nicholson 1978^[15]^	America	67/M	No	/	HTN	Yes	1	A	Autopsy	POD 8	Proximal anastomosis	No	/	/	Death
	America	51/M	No	/	HTN	Yes	/	A	Autopsy	POD 8	Proximal anastomosis	No	/	/	Death
	America	50/M	/	Abnormal	HTN, RAS	Yes	/	A	Aortogram	POD 8	/	Yes	/	/	Discharge
Goel 2012^[16]^	India	71/M	No	Abnormal	HTN, Diabetes	No	3	A	TEE	IO	Anterior wall	No	/	/	Discharge
Subramaniam 1996^[17]^	Australia	67/M	No	Normal	HTN	Yes	3	/	TEE	POD 7	Proximal anastomosis	Yes	Right atrium – femoral	Yes	Discharge
Borulu 2019^[18]^	Turkey.	60/F	No	Normal	HTN	Yes	2	A	TEE	IO	Aortic side-clamping site	Yes	/	No	Discharge
Ozasa 2003^[19]^	Japan	81/M	No	/	Diabetes	No	3	A	Ultrasound	IO	/	Yes	Femoro-femoral	Yes	Death
Tatiana 2004^[20]^	Australia	63/M	/	/	HTN, Dyslipidemia	Yes	1	B	Urgent angiography	IO	/	Yes	Implant stent-grafts	NO	Discharge
Ahmad 2023^[21]^	America	77/M	No	/	HTN, AF, PPM, Parkinson	Yes	3	A	TEE, CT-scan	POD 2	Ascending aorta	Yes	/	Yes	Discharge

AF = atrial fibrillation, ARF = anuric renal failure, BLP = bilateral leg paralysis, CA = circulatory arrest, EF = ejection fraction, F = female, HTN = hypertension, IO = Intraoperative, M = male, POD = postoperative day, PPM = post pacemaker placement, RAS = renal arterial stenosis, SAP = stable angina pectoris, TEE = transesophageal echocardiography.

**Figure 2.**
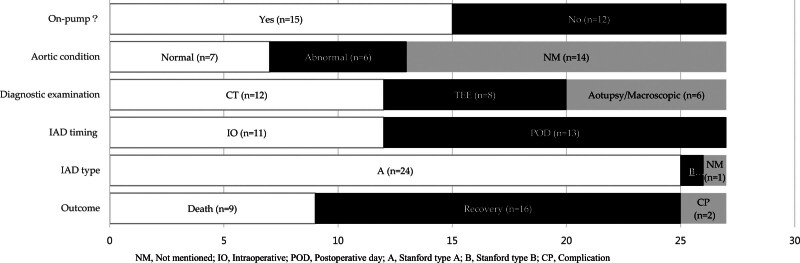
IAD-related information. IAD = Iatrogenic aortic dissection.

The 27 patients were aged 50 to 81 years, 25 (93%) males and 2 (7%) females. Among the patients, 23 (85%) were Europeans and 4 (15%) were Asians. Approximately three-fourths of the patients (74%) had unstable angina pectoris, and the others (11%) had stable angina pectoris preoperatively, whereas the characteristics of angina were not mentioned for 4 patients. A majority of patients (67%) had a history of hypertension, of whom one had concurrent diabetes and another had concurrent dyslipidemia, while the medical record was not described in 6 patients. In 8 (30%) patients, the aorta was normal preoperatively; however, an intraoperative atherosclerotic plaque with calcification at the site of the intimal dissection was observed in one of them. The aorta was abnormal in 5 (19%) patients, and the condition of the aorta was not described in 14 patients. Of these patients, 92% had more than 3 coronary artery lesions, and 16 (59%) underwent bypasses with more than 3 grafts. Figure 2 shows no significant difference in the number of on-pump (56%) and off-pump (44%) CABG procedures. In a small number of patients (37%), systolic blood pressure was maintained at 80 to 100 mm Hg during aortic side-clamping. Computed tomography (CT) and transesophageal echocardiography (TEE) were performed in 8 and 10 patients, respectively, to diagnose AD. However, in 2 patients, epiaortic ultrasound interrogation was performed to detect dissection. The report^[4]^ indicates that TEE remains a valuable tool for the prompt diagnosis of AD, and the detection of dissections is limited to the area of the distal ascending aorta and proximal aortic arch. Three patients were confirmed to have AD based on autopsy findings, and 4 cases of AD were macroscopically diagnosed intraoperatively. Interestingly, almost all IADs (93%) were Stanford type A dissections. The guidelines for diagnosing and managing patients with thoracic aortic disease categorize AD into Stanford type A and Stanford type B based on the location of the dissection and the extent of involvement.^[22]^ Moreover, in only 1 patient, Stanford type B dissection was induced during femoral artery catheterization, and the location of the dissection was not described in 1 case. Among the 27 patients, 12 (44%) had ruptured AD during operation, and 15 (56%) were complicated by AD postoperatively. Dissection originated from the aortic side-clamping site (n = 5), proximal anastomosis site (n = 8), aortic cannulation site (n = 3), and aortic root (n = 3). This resulted significantly because all breakpoint locations were in the ascending aorta. However, in 1 patient,^[8]^ AD occurred in the posterior wall of the ascending aorta, a place not associated with proximal anastomosis or side-clamping, and the origin of the AD was not described in 7 patients. Twenty (74%) patients underwent surgical treatment; among them, deep hypothermic circulatory arrest was performed in 9 patients. Conservative treatment was performed on 3 patients, including one with bilateral leg paralysis. One patient was treated with venoarterial extracorporeal membrane oxygenation. Eighteen (67%) patients were restored to health, and 9 (33%) patients died, including 3 who died before treatment.

## 4. Discussion

The data reported here appear to support that the dissection caused by CABG surgery is mostly type A (Fig. 2), which has higher mortality than type B.^[23]^ However, no relevant guidelines are currently available for managing patients undergoing CABG. This review summarized the clinical characteristics, operative information, and outcomes of 27 patients who underwent CABG.

A comprehensive preoperative assessment is crucial for formulating strategies to prevent AD. Through observations, several predisposing factors have been identified, including advanced age, a history of hypertension, aortic atherosclerosis, dilatation with thinning of the aortic wall, cystic medial necrosis, and hereditary connective tissue disorders, such as spontaneous AD.^[3,24]^ In clinical practice, a thorough evaluation of the extent of aortic calcification and the severity of coronary artery disease is of utmost importance. CT scans, ultrasound examinations, and magnetic resonance imaging (MRI) are diagnostic methods commonly used preoperatively. Additionally, experts recommend using carotid-femoral pulse wave velocity as a predictive value to assess arterial stiffness to predict cardiovascular events better, as it is easily measurable and directly reflects arterial stiffness.^[25]^ By comprehensively analyzing existing data of patients undergoing CABG surgery, we proposed a classification system to categorize patients into different risk categories. This aids in taking targeted preventive measures during surgery to minimize the risk of the occurrence of AD.

How can we choose a surgical strategy subsequently? Several reports have shown that there is no significant difference between off-pump and on-pump CABG concerning the 30-day death rate or incidence of myocardial infarction, stroke, or renal failure requiring dialysis.^[26,27]^ However, off-pump surgery is most often performed based on the individual surgeon’s judgment or habits. Although the controversy about on-pump versus off-pump CABG is likely to continue, it may be time to abandon this discussion and focus on identifying patients who benefit from either procedure.^[24]^ For relatively low-risk patients, the results of surgery are excellent with both on-pump and off-pump techniques.^[28]^ However, for higher-risk patients, it may be that alternative methods should be performed without side-clamping of the aorta or on-pump CABG should be performed to prevent this redoubtable complication.^[5]^ A detailed preoperative discussion within a multidisciplinary team should be conducted to choose the most suitable surgical technique for high-risk patients. Routine use of TEE for cardiac surgery reduces the mortality rate of IAD from 75% to 17%.^[4,29]^ Thus, TEE can be routinely used preoperatively for high-risk patients, and TEE-guided cannulation can be performed intraoperatively to prevent catastrophic complications.

ADs predominantly occur in the ascending aorta, specifically at sites such as aortic clamping, proximal anastomosis, aortic cannulation, and the aortic root. However, it is also possible for some patients to have a normal aorta. This highlights the reliance of preventing ADs on surgical techniques. Minimizing surgical trauma and performing precise surgical procedures are crucial.^[30]^ During surgery, strict control of systolic blood pressure, especially during proximal anastomosis, is a critical preventive measure. Employing a no-touch surgical approach with all-arterial grafting, avoiding aortic clamping through sequential all-arterial grafts or new-generation mechanical connectors, and occasionally resorting to aggressive aortic replacement with a prosthetic graft are recommended.^[27]^ Careful manipulation of the aorta utilizing single-sided clamping and arterial pressure control is necessary to reduce the risk of aortic injury. Ultimately, by effectively managing surgical techniques, the incidence of ADs can be significantly prevented.

Previous studies have consistently emphasized the significance of timely diagnosis and prompt intervention in patients with IAD to achieve optimal treatment outcomes.^[4,18,30,31]^ When it comes to this condition, early detection plays a vital role in preventing further complications and reducing the risk of potentially life-threatening events, such as aortic rupture or dissection. TEE is the modality of choice for the intraoperative diagnosis of AD; however, its ability to detect localized AD at the distal ascending aorta and proximal aortic arch is limited. Therefore, TEE and epiaortic ultrasonography can diagnose localized AD.^[4]^ The diagnosis of postoperative IAD poses a challenge due to its atypical presentation and lower occurrence of hemodynamic failure compared to spontaneous IAD. Patients may exhibit fewer typical symptoms, such as chest and back pain, making early detection even more difficult. However, the utilization of CT has been proven to be precise and safe for identifying IAD following open-heart surgery.^[6–8,11]^ CT imaging plays a crucial role in accurately detecting and visualizing the presence of IAD, which allows for a comprehensive assessment of the extent of dissection within the aortic wall, providing valuable information about the involvement of different segments. Furthermore, CT imaging can detect potential complications such as rupture, including its location and whether it extends into the pleural or pericardial cavities.

For treating AD, timely identification and surgical repair are crucial for successful outcomes. Although there have been reports on long-term survival without surgery in some cases, surgical treatment should still be prioritized.^[32]^ However, patients who are moribund or comatose due to established malperfusion and are at excessively high surgical risk may not be suitable candidates for surgery. In such cases, individualized medical management can be considered based on the outcomes of local centers. Comprehensive risk assessment of patients should consider various factors, including comorbidities and complications related to AD, rather than relying solely on single factors such as age.^[33]^CC Furthermore, establishing dedicated specialist units can provide more specialized treatment management, enhance the integration and collaboration of medical resources, and ultimately achieve better treatment outcomes. For cases without monitoring, the cooling time of patients should be at least 50 minutes or until the nasopharyngeal temperature drops below 18°C. A high-volume multidisciplinary thoracic aortic surgery team can achieve treatment outcomes similar to elective proximal aortic surgery in repairing acute type A AD.^[34]^ For hemi-arch or total arch repair, the completion of open distal anastomosis is guided by cerebral electroencephalogram monitoring under deep hypothermic circulatory arrest, with controlled cooling time.^[17]^ These specialist units can offer comprehensive medical services, including diagnosis, surgical treatment, and postoperative care, ensuring patients receive the most optimal treatment plans and nursing support. This approach also contributes to improving disease diagnosis and management, as well as promoting medical research and academic exchange.

## 5. Suggestions

Even patients with a normal aorta during preoperative examination may still experience IAD, and therefore, we must not take it lightly. It is necessary to perform TEE before and after aortic manipulation. To minimize the risk of inducing IAD during coronary artery bypass surgery, we need to adhere to the following measures. First, the accuracy and reliability of monitoring devices should be ensured, and monitoring results should be recorded in real-time. Second, individualized treatment plans should be developed based on the patient’s condition and surgical needs. Third, anesthesiologists should adjust the type and dosage of medications according to the patient’s condition and the type of surgery. Fourth, close collaboration among surgeons, anesthesiologists, and intensive care nurses is essential to develop strategies and plans for regulating vital signs. Effective communication and coordination should be ensured. Fifth, the patient’s vital signs should be monitored and observed for prompt adjustment of treatment plans based on changes and sharing information with the entire team. The patient’s condition and treatment progress should be continuously assessed and documented. By implementing these measures, we can effectively reduce the risk of IAD during coronary artery bypass surgery and ensure patient safety.

Many questions regarding the lack of a reliable scoring system that can be used to assess a patient’s risk level for predicting the incidence of IAD remain unanswered. Therefore, more clinical trials are needed to develop such a scoring scale. This would be beneficial for developing individualized treatment measures and improving patient prognosis.

**Figure 3.**
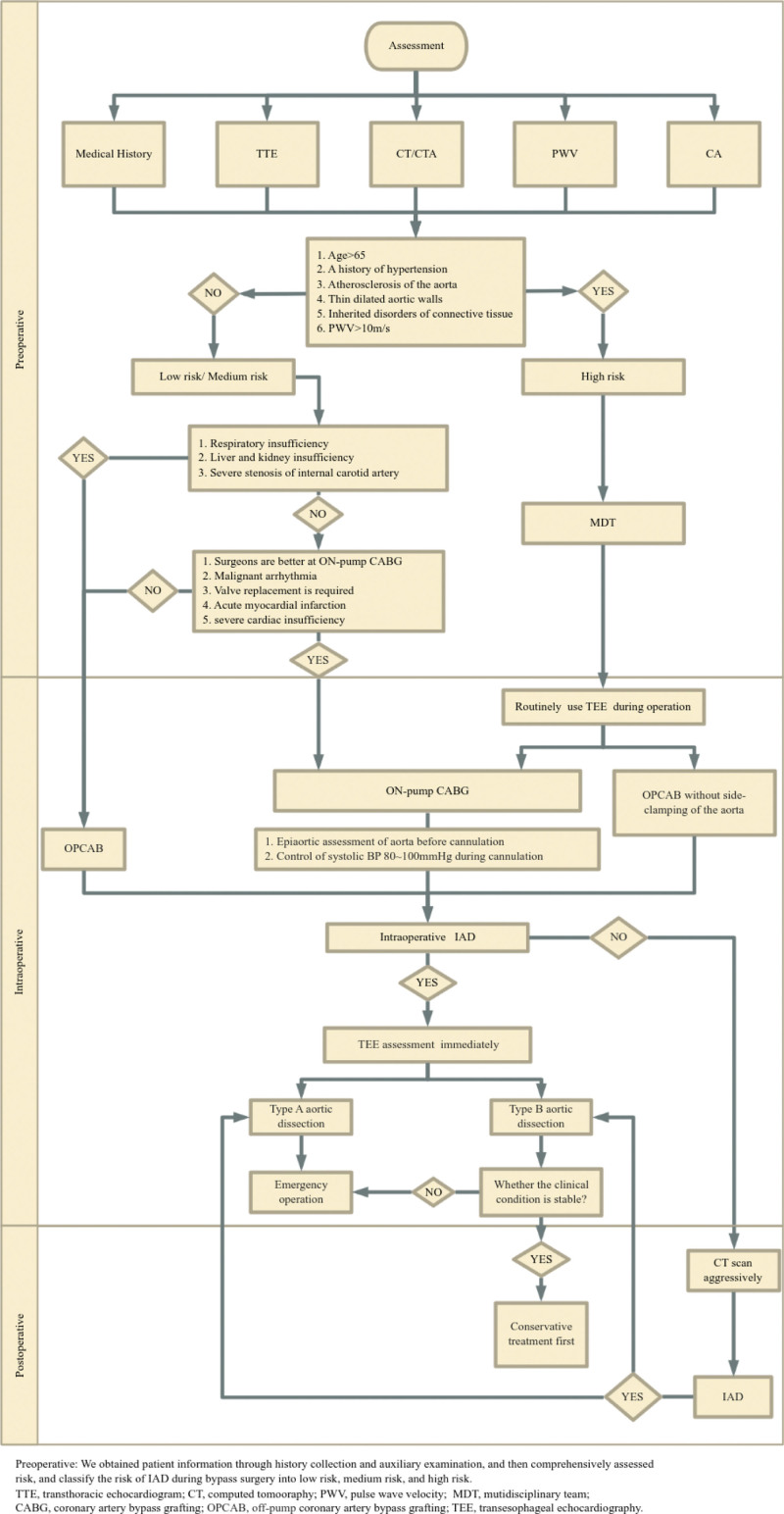
General process to reduce IAD of patients undergoing CABG surgery.

By incorporating the practices above and recommendations, we have developed a recommended management protocol (Table 2) and flowchart (Fig. 3) for patients undergoing CABG surgery.

**Table 2:** Recommended methods and management of patients undergoing CABG surgery.

Preoperative examination and treatment	
Medical history	Collect patient information, including age, sex, and medical history.
CT/CTA	Assessing the aorta for normality.
CA	Location and extent of coronary artery occlusion.
PWV	Assessment of the condition of arteriosclerosis.
TTE	Evaluation of cardiovascular structure/function and volume status.
Medication	Control blood pressure and improve cardiac function.
Preoperative assessment	
Risk classification	As shown in (Figure 3).
MDT	Surgery, anesthesia, ultrasonics, radiology, critical care, etc.
Preventative methods	
On-pump or off-pump	Individualized selection of whether extracorporeal circulation or not.
Equipment	Routinely use TEE during operation, especially with a high risk of IAD.
	Epiaortic assessment of the aorta before cannulation by using ultrasound.
Monitoring	Control of systolic BP 80~100mmHg during cannulation.
Arteria femoral	Arteria femoral should be ready for rescue use.
Surgical procedures	Minimal and careful use of side-clamping.
	For OPCAB, avoid clamp use and favor control of pulsatility.
Postoperative	CT scan aggressively.
Management of IAD	
	A high-volume multidisciplinary thoracic aortic surgery team performed the operation.
	Transesophageal echocardiographic and invasive hemodynamic monitoring
	Deep hypothermic circulatory arrest.

CA = coronary arrest, CABG = coronary artery bypass grafting, CT = computed tomography, IAD = Iatrogenic aortic dissection, MDT = mutidisciplinary team, OPCAB = off-pump coronary artery bypass grafting, PWV = pulse wave velocity, TTE = transthoracic echocardiogram.

## 6. Conclusions

The occurrence of iatrogenic aortic dissection as a result of coronary artery bypass surgery depends on the patient’s condition, the surgeon’s skill, and the teamwork involved. The association with cardiopulmonary bypass needs further investigation for confirmation. It is crucial to take preventive measures to minimize the occurrence of iatrogenic aortic dissection (IAD) during coronary artery bypass surgery, as these dissections are often hazardous type A dissections.

## Acknowledgments

We appreciate the assistance provided by the company of Editage in terms of language modifications.

## Author contributions

**Conceptualization:** Yuntai Yao.

**Data curation:** Xi Yuan, Huaqiu Chen, Qiqing Lan, Wen Wu, Yuntai Yao.

**Formal analysis:** Xi Yuan.

**Funding acquisition:** Yuntai Yao.

**Software:** Huaqiu Chen.

**Writing – original draft:** Xi Yuan.

**Writing – review & editing:** Yanting Sun, Yuntai Yao.

## References

[1] Biancari F Pettinari M Mariscalco G. Outcome after surgery for iatrogenic acute type A aortic dissection. J Clin Med. 2022;11:6729. 36431205 10.3390/jcm11226729 PMC9696328

[2] Ram H Dwarakanath S Green AE. Iatrogenic aortic dissection associated with cardiac surgery: a narrative review. J Cardiothorac Vasc Anesth. 2021;35:3050–66. 33008721 10.1053/j.jvca.2020.07.084

[3] Chavanon O Carrier M Cartier R. Increased incidence of acute ascending aortic dissection with off-pump aortocoronary bypass surgery? Ann Thorac Surg. 2001;71:117–21. 11216729 10.1016/s0003-4975(00)02136-6

[4] Assaad S Geirsson A Rousou L. The dual modality use of epiaortic ultrasound and transesophageal echocardiography in diagnosing intraoperative iatrogenic type-a aortic dissection. J Cardiothorac Vasc Anesth. 2013;27:326–8. 22129791 10.1053/j.jvca.2011.09.026

[5] Li J Guan X Gong M. Iatrogenic acute aortic dissection induced by off-pump coronary artery bypass grafting: a case report and review of the literature. Medicine (Baltimore). 2017;96:e9206. 29390466 10.1097/MD.0000000000009206 PMC5758168

[6] Tabry I Costantini E. Acute aortic dissection early after off-pump coronary surgery: true frequency underestimated? Tex Heart Inst J. 2009;36:462–7. 19876430 PMC2763454

[7] De Smet J Stefanidis C. Acute aortic dissection after off-pump coronary artery surgery. Eur J Cardiothorac Surg. 2003;24:315–7. 12895634 10.1016/s1010-7940(03)00296-3

[8] Takahashi B Uchino M Takeuchi Y. Acute type A aortic dissection with a tear in the posterior wall of the ascending aorta early after off-pump coronary artery bypass grafting. Gen Thorac Cardiovasc Surg. 2021;69:870–3. 33201384 10.1007/s11748-020-01547-4

[9] Ram H Weaver AR Dorfling J. Iatrogenic aortic intramural hematoma: guidance to intraoperative decision making: a case report. A A Pract. 2020;14:e01191. 32224700 10.1213/XAA.0000000000001191

[10] Pappas D Hines GL Gennaro M. Delayed iatrogenic aortic dissection from coronary bypass managed with extraanatomic bypass. J Thorac Cardiovasc Surg. 1998;115:947–9. 9576234 10.1016/S0022-5223(98)70379-4

[11] Archer A Choyke P Zeman R. Aortic dissection following coronary artery bypass surgery: diagnosis by CT. Cardiovasc Intervent Radiol. 1986;9:142–5. 3089621 10.1007/BF02577924

[12] Cottrell DJ Cornett ES Seifer MS. Diagnosis of an intraoperative aortic dissection by transesophageal echocardiography during routine coronary artery bypass grafting surgery. Anesth Analg. 2003;97:1254–6. 14570632 10.1213/01.ANE.0000083640.41295.97

[13] Bilgutay A Garamella J Danyluk M. Retrograde aortic dissection occurring during cardiopulmonary bypass. Successful repair and concominant subclavian-to-coronary artery vein bypass. JAMA. 1976;236:465–8. 1084428

[14] Magishi K Izumi Y Shimizu N. Spinal cord infarction due to aortic dissection during coronary artery bypass grafting under percutaneous cardiopulmonary support. Kyobu Geka. 2010;63:1137–40. 21174663

[15] Nicholson WJ Crawley IS Logue RB. Aortic root dissection complicating coronary bypass surgery. Am J Cardiol. 1978;41:103–7. 304660 10.1016/0002-9149(78)90139-x

[16] Goel S Majhi S Panigrahi B. Intraoperative detection of aortic dissection after off-pump coronary artery bypass grafting. J Cardiothorac Vasc Anesth. 2012;26:e11–12. 22155169 10.1053/j.jvca.2011.10.006

[17] Subramaniam P Skillington P. Acute aortic dissection as a complication of coronary artery surgery. Aust N Z J Surg. 1996;66:498–500. 8678885 10.1111/j.1445-2197.1996.tb00793.x

[18] Borulu F Erkut B. Acute dissection of the ascending aorta as a rare complication of aortocoronary bypasses surgery: a case report. J Tehran Heart Cent. 2019;14:191–4. 32461761 PMC7231685

[19] Ozasa H Toyota K Uchida H. Acute aortic dissection during vertical displacement of the heart in off-pump coronary artery bypass grafting (OPCAB). J Anesth. 2003;17:274–6. 14625717 10.1007/s00540-003-0185-5

[20] Fleck T Wisser W Cejna M. Complicated acute aortic dissection type B caused by femoral cannulation for endoscopic coronary artery bypass surgery. J Endovasc Ther. 2004;11:80–3. 14748625 10.1177/152660280401100110

[21] Mahdi A Akkawi AR Mahdi M. The silent threat: a case of iatrogenic asymptomatic aortic dissection post coronary artery bypass grafting. Cureus. 2023;15:e41035. 37519582 10.7759/cureus.41035 PMC10374978

[22] Hiratzka LF Bakris GL Beckman JA.; American College of Cardiology Foundation/American Heart Association Task Force on Practice Guidelines. 2010 ACCF/AHA/AATS/ACR/ASA/SCA/SCAI/SIR/STS/SVM guidelines for the diagnosis and management of patients with thoracic aortic disease: a report of the American College of Cardiology Foundation/American Heart Association Task Force on Practice Guidelines, American Association for Thoracic Surgery, American College of Radiology, American Stroke Association, Society of Cardiovascular Anesthesiologists, Society for Cardiovascular Angiography and Interventions, Society of Interventional Radiology, Society of Thoracic Surgeons, and Society for Vascular Medicine. Circulation. 2010;121:e266–369. 20233780 10.1161/CIR.0b013e3181d4739e

[23] Obel LM Lindholt JS Lasota AN. Clinical characteristics, incidences, and mortality rates for type A and B aortic dissections: a Nationwide Danish population-based cohort study from 1996 to 2016. Circulation. 2022;146:1903–17. 36321467 10.1161/CIRCULATIONAHA.122.061065

[24] Blackstone EH Sabik JF 3rd. Changing the discussion about On-Pump versus Off-Pump CABG. N Engl J Med. 2017;377:692–3. 28813212 10.1056/NEJMe1706220

[25] Van Bortel LM Laurent S Boutouyrie P.; Artery Society. Expert consensus document on the measurement of aortic stiffness in daily practice using carotid-femoral pulse wave velocity. J Hypertens. 2012;30:445–8. 22278144 10.1097/HJH.0b013e32834fa8b0

[26] Lamy A Devereaux PJ Prabhakaran D.; CORONARY Investigators. Off-pump or on-pump coronary-artery bypass grafting at 30 days. N Engl J Med. 2012;366:1489–97. 22449296 10.1056/NEJMoa1200388

[27] Diegeler A Börgermann J Kappert U.; GOPCABE Study Group. Off-pump versus on-pump coronary-artery bypass grafting in elderly patients. N Engl J Med. 2013;368:1189–98. 23477657 10.1056/NEJMoa1211666

[28] Taggart DP Altman DG. Off-pump vs. on-pump CABG: are we any closer to a resolution? Eur Heart J. 2012;33:1181–3. 22065846 10.1093/eurheartj/ehr374

[29] Hwang HY Jeong DS Kim KH. Iatrogenic type A aortic dissection during cardiac surgery. Interact Cardiovasc Thorac Surg. 2010;10:896–9. 20299447 10.1510/icvts.2009.231001

[30] Stamou SC Kouchoukos NT Hagberg RC. Differences in clinical characteristics, management, and outcomes of intraoperative versus spontaneous acute type A aortic dissection. Ann Thorac Surg. 2013;95:41–5. 23084415 10.1016/j.athoracsur.2012.08.050

[31] Etuk AS Odigwe CI Singu S. Incidental finding of thoracic aortic dissection in a patient post-coronary artery bypass graft surgery. Cureus. 2023;15:e40443. 37456414 10.7759/cureus.40443 PMC10349285

[32] Howard DP Banerjee A Fairhead JF.; Oxford Vascular Study. Population-based study of incidence and outcome of acute aortic dissection and premorbid risk factor control: 10-year results from the Oxford Vascular Study. Circulation. 2013;127:2031–7. 23599348 10.1161/CIRCULATIONAHA.112.000483 PMC6016737

[33] Bonser RS Ranasinghe AM Loubani M. Evidence, lack of evidence, controversy, and debate in the provision and performance of the surgery of acute type A aortic dissection. J Am Coll Cardiol. 2011;58:2455–74. 22133845 10.1016/j.jacc.2011.06.067

[34] Andersen ND Ganapathi AM Hanna JM. Outcomes of acute type a dissection repair before and after implementation of a multidisciplinary thoracic aortic surgery program. J Am Coll Cardiol. 2014;63:1796–803. 24412454 10.1016/j.jacc.2013.10.085 PMC4159705

